# Integrated Analysis of Dysregulated ncRNA and mRNA Expression Profiles in Humans Exposed to Carbon Nanotubes

**DOI:** 10.1371/journal.pone.0150628

**Published:** 2016-03-01

**Authors:** Anna A. Shvedova, Naveena Yanamala, Elena R. Kisin, Timur O. Khailullin, M. Eileen Birch, Liliya M. Fatkhutdinova

**Affiliations:** 1 Exposure Assessment Branch/HELD/NIOSH/CDC, Morgantown, WV - 26505, United States of America; 2 Department of Physiology and Pharmacology, West Virginia University, Morgantown, WV -26505, United States of America; 3 NIOSH/CDC, 4676 Columbia Parkway, Cincinnati, OH - 45226, United States of America; 4 Department of Hygiene and Occupational Health, Kazan State Medical University, ul. Butlerova 49, Kazan, 420012 Russia; Harbin Medical University, CHINA

## Abstract

**Background:**

As the application of carbon nanotubes (CNT) in consumer products continues to rise, studies have expanded to determine the associated risks of exposure on human and environmental health. In particular, several lines of evidence indicate that exposure to multi-walled carbon nanotubes (MWCNT) could pose a carcinogenic risk similar to asbestos fibers. However, to date the potential markers of MWCNT exposure are not yet explored in humans.

**Methods:**

In the present study, global mRNA and ncRNA expression profiles in the blood of exposed workers, having direct contact with MWCNT aerosol for at least 6 months (n = 8), were compared with expression profiles of non-exposed (n = 7) workers (e.g., professional and/or technical staff) from the same manufacturing facility.

**Results:**

Significant changes in the ncRNA and mRNA expression profiles were observed between exposed and non-exposed worker groups. An integrative analysis of ncRNA-mRNA correlations was performed to identify target genes, functional relationships, and regulatory networks in MWCNT-exposed workers. The coordinated changes in ncRNA and mRNA expression profiles revealed a set of miRNAs and their target genes with roles in cell cycle regulation/progression/control, apoptosis and proliferation. Further, the identified pathways and signaling networks also revealed MWCNT potential to trigger pulmonary and cardiovascular effects as well as carcinogenic outcomes in humans, similar to those previously described in rodents exposed to MWCNTs.

**Conclusion:**

This study is the first to investigate aberrant changes in mRNA and ncRNA expression profiles in the blood of humans exposed to MWCNT. The significant changes in several miRNAs and mRNAs expression as well as their regulatory networks are important for getting molecular insights into the MWCNT-induced toxicity and pathogenesis in humans. Further large-scale prospective studies are necessary to validate the potential applicability of such changes in mRNAs and miRNAs as prognostic markers of MWCNT exposures in humans.

## Introduction

Nanotechnology presents new opportunities to create superior materials with novel applications. At present, nanomaterial (NM) containing products are available in U.S. and worldwide markets including coatings, computers, clothing, cosmetics, sports equipment and medical devices. NMs have experienced rapid and substantial industrial growth globally. In fact, this manufacturing growth is accompanied by questions regarding potential risks to human health. Meanwhile, epidemiological studies documented the effect of air pollution on pulmonary, and cardiovascular morbidity, and mortality. Toxicological literature describes the translocation of carbonaceous NM across the alveolar-capillary barrier into the circulatory and lymphatic systems, providing access to most organs in the body [1–5].

Multiwalled carbon nanotubes (MWCNTs) are allotropes of carbon that are formally viewed as a tubular structure rolled up from a graphene sheet. The cylindrical nature of MWCNT, exceptional mechanical strength and intrinsic physico-chemical properties are in use for a number applications in electronics, optics, materials science, polymer chemistry and nanocomposites[6, 7]. MWCNTs have diameters in the range of 2−10 nm for the innermost tubular layer and an additional thickness of ∼0.7 nm for every extra layer. Different chiral vectors, which describe how the graphene sheets are rolled up, result in MWCNTs with different electronic structure. They are either semiconducting or metallic [8]. Because of the high aspect ratio and good conductivity, MWCNTs are especially promising as conductive fillers for making polymeric composites with high-strength and electric-shielding properties [9–11].

Enlarged volume of MWCNTs manufacturing has raised concerns over potential risks of adverse health effects in humans since there is the likelihood of rising with time. Due to unique physical and chemical features, MWCNTs may have quite unusual pulmonary responses [12–14]. Several studies have focused on respiratory toxicity, particle retention and low pulmonary clearance observed in the lungs of exposed animals resembling malaise effects of asbestos [2, 12, 15]. The high aspect ratio, size distribution and biopersistence of MWCNTs is likely associated with pulmonary inflammation, damage, fibrogenicity along with their ability to potentiate tumorigenesis in rodents poses a major safety concern. Recently, the International Agency for Research on Cancer (IARC) has classified MWCNT-7 (Mitsui Ltd., Japan) to a Category 2B: possibly carcinogenic to humans [16]. Potential human exposures to NMs or their mixtures include workers exposed during the production and use of NM, general population exposure from releases into the environment and human exposure during the use of commercially available products. However, to date the potential markers of MWCNT exposure and their plausible adverse health outcomes are not yet explored in humans.

The non-coding RNAs (ncRNA), especially long non-coding RNAs (lncRNA) and micro RNAs (miRNA) have emerged as crucial regulators of various biological processes by interfering with gene expression. The miRNAs, a class of small single-stranded ncRNAs (≈22 nt), negatively regulate gene expression through partial base-pairing with target genes or coding messenger RNAs (mRNA). In contrast, lncRNAs, a set of diverse ncRNAs, have been shown to regulate gene expression through various mechanisms, including complementary binding to mRNAs in the form of cis-antisense lncRNAs, modulating transcription factors[17–21]. Despite attracting a great deal of interest in several human disease conditions including cancer [22–24], systematic and integrative analyses of different types of RNA molecules have been very rare in the field of nanotoxicology. Thus an integrated analysis involving both coding-noncoding RNA molecules is critical for determining and understanding the regulatory networks and molecular mechanisms underlying MWCNT induced biological responses and toxicity in humans. In this paper, we determined and evaluated changes in global non-coding lncRNAs/miRNAs and coding mRNA expression profiles, through genome-wide approaches, in the whole blood samples of workers exposed to the MWCNT aerosols from manufacturing facilities vs non-exposed individuals. The changes in the miRNA/mRNA expression profiles were correlated with known adverse cardiopulmonary outcomes associated with MWCNT exposures in rodents. Functional and pathway enrichment analysis was performed to identify genes and pathways that may contribute to MWCNT-induced outcomes in humans. Furthermore, we performed an integrated analysis of ncRNA-mRNA targets based on their interactions and positional information to suggest and generate a regulatory network of interactions between lncRNAs, miRNAs and mRNAs in MWCNT-exposed workers. The changes in coding-noncoding RNAs identified in the present study may have a promising potential as relevant blood markers for monitoring MWCNT exposure in humans.

## Materials and Methods

### Exposure Assessment

As part of this study, workplace/occupational exposure assessments were conducted at the Nanotech Center Ltd. (Tambov, Russia) MWCNT manufacturing facility. Air sampling was performed during the main operations (harvesting, disintegration, packaging, laboratory handling) throughout the production process. Samples were collected on ultraclean, 25-mm quartz-fiber filters (Pallflex Tissuquartz™, 2500 QAT-UP) using PU-4E pumps (Khimko JSC, Moscow, Russia) operating at 2–4 L min^-1^. A total of 270 L of air was sampled. After sample collection, the quartz filters were analyzed for elemental carbon (EC) according to NIOSH Method 5040 [3], recommended by NIOSH for assessing airborne exposure to CNTs and carbon nanofibers (CNFs)[25]. The analysis is based on a thermal-optical technique for organic and elemental carbon (OC and EC), with EC being a measure of the CNT/CNF mass. Bulk CNT samples also were analyzed to obtain their thermal profiles. As discussed previously [25, 26], CNT (and CNF) aerosols contain micrometer-size agglomerates. The automated OC-EC split that is normally assigned in the 5040 analysis is helpful to OC-EC speciation for complex carbonaceous aerosols, but the optical technique used for speciation was intended for fine/ultrafine aerosols. Because sample transmittance (absorbance) has a particle-size dependence, with larger particles having less light attenuation, the auto-split may underestimate CNT mass due to the relatively large particle size. This problem can be avoided through manual assignment of the split, based on the results for bulk materials (microgram amounts) and background samples. In this study, environmental background EC was negligible and posed no interference in the CNT measurement. In addition, the CNT materials contained little OC, if any, and they were fully oxidized during the oxidative mode (920°C maximum) of the analysis. As such, quantification of the CNT filter content was straightforward.

Background samples for EC were collected in the same manner at each facility to account for any external sources of EC, not related to MWCNT manufacturing and handling. To confirm the presence of MWCNTs, air samples were also collected on 37-mm diameter mixed cellulose ester (MCE) filters (0.8 μm pore size; SKC, Inc., USA) for analysis by transmission electron microscopy (TEM). The TEM samples were analyzed on a JEOL2100F TEM (JEOL USA, Inc., Peabody, MA, USA) using a modified NMAM 7402 [27]. These modifications consist mostly of eliminating all steps necessary to positively identify asbestos [26]. The modifications mainly relate to counting CNT/CNF particles, which, unlike asbestos fibers, occur mainly as micrometer-sized agglomerates. Three 3 mm, copper TEM grids from each sample were examined at low magnification to determine loading and sample preparation quality. Elemental spectra were also obtained using energy dispersive x-ray analysis (EDXA).

### Study subjects and data collection

The physico-chemical properties of MWCNTs manufactured and/or used in the Nanotech Center Ltd. facility are listed in S1 Table. A total of 8 workers exposed to MWCNTs and 7 non-exposed controls from the same facility were recruited to take part in this study. The classification of exposed workers and non-exposed controls was based on work duties and confirmed by the presence and absence, respectively, of MWCNTs in personal breathing zones (PBZ) samples (S2 Table). The non-exposed controls were selected from workers at the same sites as the exposed workers, but who did not handle MWCNTs. The Institutional Review Board of Kazan State Medical University, Kazan, Russia, approved this study under the protocol №14 dated December 26, 2011. Informed consent was obtained from each of the subjects after a detailed explanation of the nature and possible consequences of the study by the interviewer on the day of the personal interview. After a written informed consent was obtained from individual participants, the subjects were interviewed in person using a structured questionnaire and health examinations. Demographic and health data included age, gender, pernicious habits, work experience, and history of disease. Exposure group inclusion criteria were as follows: age 18–60 y; no less than 6 months work experience with MWCNT exposure (based on hygienic workplace investigation); no acute or chronic respiratory, cardiovascular or connective tissue diseases, no recent traumas, arterial pressure no more than 140/90 mmHg.; no general medical contraindications to the blood collection. In order to eliminate the potential effects of other confounding factors such as sex and smoking, in addition to MWCNTs, males who are non-smokers were considered for performing detailed whole blood gene expression profiling studies. While the non-exposed group (n = 7) was comprised of individuals in the age range 20–30 yrs old, the MWCNT exposed group (n = 8) involved workers with ages 23–60 yrswho spent an average of ~ 6–24 months working in the facility.

### Blood sample collection and storage

Peripheral whole blood (~2–2.5 mL) from venipuncture was dispensed into the PAXgene™ Blood RNA Tubes (Qiagen). The sample was gently inverted and frozen at -80°C within two hours of collection.

### Total RNA isolation and quality assessments

RNA was extracted from whole blood using the PAXgene™ Blood RNA System Kit following manufacturer's guidelines. Briefly, the samples were removed from -80°C and incubated at room temperature for 2 hours to ensure complete lysis. Following lysis the tubes were centrifuged for 10 min at 5,000 × g, the supernatant decanted and 500 μL of RNase-free water added to the pellet. After washing with 500 μl RNase-free water, the pellet was dissolved in 350 μl resuspension buffer and incubated with 300 μl binding buffer and 40 μl proteinase K for 10 min at 55°C in a shaker-incubator. The lysate was transferred into a PAXgene shredder spin column and centrifuged (at 18,000 g for 3 min). The flow-through fraction was mixed with 350 μl ethanol and transferred to a PAXgene RNA spin column. After washing the column with washing buffer 1, samples were incubated with 10 μl of DNase I for 15 min. PAXgene RNA spin columns were washed with washing buffer and RNA eluted with 40 μl elution buffer. The RNA yield was estimated by measuring absorbance at 260 nm in a NanoDrop ND-1000 spectrophotometer (Thermo Fisher Scientific, Wilmington, DE, USA). RNA purity was calculated from the ratio of absorbance at 260 nm and 280 nm, and RNA integrity was assessed by standard denaturing agarose gel electrophoresis.

### LncRNA & mRNA labeling, array hybridization and data analysis

Human LncRNA microarray V3.0, designed by ArrayStar Inc, was employed to perform global profiling of human LncRNAs (~30,586) and protein-coding (~26,109) transcripts. Sample labeling and array hybridization were performed according to the Agilent One-Color Microarray-Based Gene Expression Analysis protocol (Agilent Technology) with minor modifications. Briefly, mRNA was purified from total RNA after removal of rRNA (mRNA-ONLY™ Eukaryotic mRNA Isolation Kit, Epicentre). Then, each sample was amplified and transcribed into fluorescent cRNA along the entire length of the transcripts without 3’ bias utilizing a random priming method. The labeled cRNAs were purified by RNeasy Mini Kit (Qiagen). The concentration and specific activity of the labeled cRNAs (pmol Cy3/μg cRNA) were measured by NanoDrop ND-1000. 1 μg of each labeled cRNA was fragmented by adding 5 μl 10 × Blocking Agent and 1 μl of 25 × Fragmentation Buffer, then heated the mixture at 60°C for 30 min, finally 25 μl 2 × GE Hybridization buffer was added to dilute the labeled cRNA. 50 μl of hybridization solution was dispensed into the gasket slide and assembled to the LncRNA expression microarray slide. The slides were incubated for 17 hours at 65°C in an Agilent Hybridization Oven. The hybridized arrays were washed, fixed and scanned using the Agilent DNA Microarray Scanner (part number G2505C).

Agilent Feature Extraction software (version 11.0.1.1) was used to analyze acquired array images. Quantile normalization and subsequent data processing were performed with using the GeneSpring GX v11.5.1 software package (Agilent Technologies). The LncRNAs and mRNAs showing expression changes of at least 1.5-fold in MWCNT-exposed group compared to non-exposed controls and having p-values of less than or equal to 0.05 (p ≤ 0.05, t-test) were considered significantly differentially expressed and were considered for further analysis. Hierarchical Clustering was performed using the Agilent GeneSpring GX software (version 11.5.1). All lncRNA and mRNA expression data were deposited to NCBI's Gene Expression Omnibus and is accessible via accession number (ID: GSE73830). To allow for the identification and determination of MWCNT related exposure markers, the samples corresponding to the MWCNT exposure group were further grouped into two categories: (i) overall exposure group—consisting all MWCNT exposed workers and (ii) high exposure group—workers exposed to MWCNT lung burdens of > 9μg per day (Sample IDs:- TB1, TB2 and TB14).

### miRNA sequencing and expression profiling

Total RNA of each sample was used to prepare the miRNA sequencing library which includes the following steps: 1) 3'-adapter ligation with T4 RNA ligase 2 (truncated); 2) 5'-adapter ligation with T4 RNA ligase; 3) cDNA synthesis with RT primer; 4) PCR amplification; 5) extraction and purification of ~135–155 bp PCR amplified fragments (correspond to ~15–35 nt small RNAs) from the PAGE gel. After the completed libraries were quantified with Agilent 2100 Bioanalyzer, the DNA fragments in the libraries were denatured with 0.1M NaOH to generate single-stranded DNA molecules, captured on Illumina flow cells, amplified in situ and finally sequenced for 36 cycles on Illumina HiSeq 2000 according to the manufacturer’s instruction.

After sequencing images were generated, image analysis and base calling was performed using Off-Line Basecaller software (OLB V1.8.0). Subsequently, 3’ adapter sequences were trimmed from clean reads (reads that passed Solexa CHASTITY quality filter) and the reads shorter than 15nt were discarded. Then the 3’-adapter-trimmed-reads (> = 15nt) were aligned to the latest known human reference miRNA precursor set (Sanger miRBase 20) using Novoalign (v2.07.11). Reads (counts < 2) were discarded when calculating the miRNA expression. In order to characterize the isomiR variability, any sequence that matched the miRNA precursors in the mature miRNA region ±4nt (no more than one mismatch) were accepted as mature miRNA isomiRs, which were grouped according to the 5-prime (5p) or 3-prime (3p) arm of the precursor hairpin.

### Bioinformatics Analysis and Approaches

#### Identification and Prediction of LncRNA-mRNA/-miRNA interactions and miRNA-mRNA Target pairs

The microRNA Target Filter of Ingenuity Pathway Analysis (IPA) software was used to select only the miRNA‐mRNA target interactions that were experimentally demonstrated or predicted with the highest level of confidence. Based on experimental data and the information provided by the Ingenuity Knowledge Base, this tool helps users to quickly prioritize mRNA targets using algorithms miRecords[28], TargetScan[29], and TarBase[30]. For miRNAs that were up-regulated in MWCNT-exposed workers compared to non-exposed group, we searched for mRNA target pairs that were down-regulated, and vice versa.

The LncRNABase of StarBase v2.0 [31, 32] was used to identify and predict miRNA interactions and/or targets on long non-coding RNAs. This database provides experimentally verified and computationally predicted miRNA recognition elements (MREs) or targets on human lncRNAs. Further the lncRNAs were classified into different subgroups and analyzed according to their various interaction mechanisms with mRNAs. The functional relationships between nearby coding genes and the lncRNAs with enhancer-like functions and large intervening noncoding RNAs (lincRNAs) were screened and identified by mapping their genomic position using GENCODE and its psuedogene, RefSeq and UCSC annotations of the human genes[33–37], as well as previous studies by Rinn’s group[38–40].

#### Core Analysis using IPA

To identify and reveal biological pathways and signaling networks that are significantly perturbed in workers potentially exposed to MWCNTs, Ingenuity Pathway Analysis (IPA, ver. 8.6; Redwood City, CA; www.ingenuity.com) was performed on differentially expressed (p ≤ 0.05) mRNAs, miRNAs and their co-expressed gene targets with consistent expression in mRNA profiles. Tab delimited text files containing gene IDs, expression data, and t-test p-values were uploaded into IPA for conducting a core analysis. A fold change cutoff of 1.5, as well as a p-value cut-off of 0.05 was set to identify genes and/or mRNA targets whose expression was significantly differentially regulated in each case. Score rankings for the top molecular/cellular/biological functions, diseases, toxicology functions, and IPA-identified gene signaling networks (GSN) were calculated using IPA-generated negative logarithm p-values i.e., -log10(p-value) and associated Z- and network scores. The estimated Fischer’s exact p-values, pathway- and network-activation scores will reflect the probability or likelihood of genes occurring in a given pathway/biofunction/disease/network versus others is based on pure chance or not.

#### Pathway enrichment analysis of mRNAs and miRNA-mRNA target pairs

Kyoto Encyclopedia of Genes and Genomes (KEGG) pathway enrichments on differentially expressed genes was performed using Database for Annotation, Visualization and Integrated Discovery (DAVID) v6.7 software [41, 42]. DIANA-miRPath v2.0 (http://www.microrna.gr/miRPathv2), a web-based computational tool, was used to analyze and identify molecular pathways associated with the putative targets of miRNAs [43, 44]. This program predicts miRNA targets with high accuracy based on experimentally supported miRNA-mRNA interactions from TarBase and also the DIANA-microT-CDS algorithm that considers the evolutional conservation of miRNA-binding sites. An enrichment analysis, depicting the target genes of multiple miRNAs in Kyoto Encyclopedia of Genes and Genomes (KEGG) pathways was performed. KEGG (www.genome.jp/kegg/kegg_ja.html) is a well-known publicly accessible knowledgebase, composed of manually curated 364,925 pathways that cover a wide range of metabolic, genetic, environmental, and cellular processes and human diseases. A p-value cutoff of < 0.05 was used for identifying significantly perturbed KEGG pathways. We also utilized the gene filtering option of the DIANA-miRPath to filter for miRNA targets that had consistent expression in mRNA expression profiles in the blood and a similar pathway enrichment analysis using Tarbase, as described above, was performed using only the expressed subset of genes.

#### Quantitative Real Time PCR Validation of mRNAs and miRNAs

The quantification of the six selected mRNA transcripts (CCND1, CCND3, E2F2, SOD1, TGFβ1, EGFR and MUC1) was performed by relative quantitative real-time RT-PCR with TaqMan real-time RT-PCR assays and 7900HT Fast Real-time PCR system (Applied Biosystems, Foster City, CA). Forward and reverse primers were designed using Primer Premier 5.0 software. cDNAs were generated from 1.5 μg of total RNA using SuperScript III First-Strand Synthesis SuperMix kit (InVitrogen, Carlsbad, CA). The resulting cDNA was subjected to 40 cycle PCR amplification. Each thermocycling reaction used 10 μl total volume containing 2 μl cDNA, 1.2 μl primers, 1.8 μl ddH_2_O and 5 μl PCR Master Mix. All samples corresponding to the selected mRNAs were normalized to Glyceraldehyde-3-phosphate dehydrogenase (GAPDH). All of the reactions were run in triplicate. The mean value in each triplicate was used to calculate relative concentrations of each mRNA (ΔCt = Ct mean mRNAs−Ct mean GAPDH). Expression fold changes were calculated using 2^−ΔΔCt^ method (ΔΔCt =  ΔCt _MWCNT-exposed_ mRNAs − ΔCt _unexposed_ mRNAs). The differences in the level of mRNA expression between MWCNT exposed and unexposed subjects were analyzed using the Student’s t-test and a p-value of < 0.05 was considered as statistically significant. Correlation between the microarray and qPCR results for the selected genes was performed using Pearson’s correlation, and the statistical significance of the correlations was then determined by estimating the test-statistic—t* and *P*-value by referring to a *t-*distribution with *n*-2 degrees of freedom.

## Results

### TEM and EC analyses indicate presence of airborne CNT

**Fig 1:**
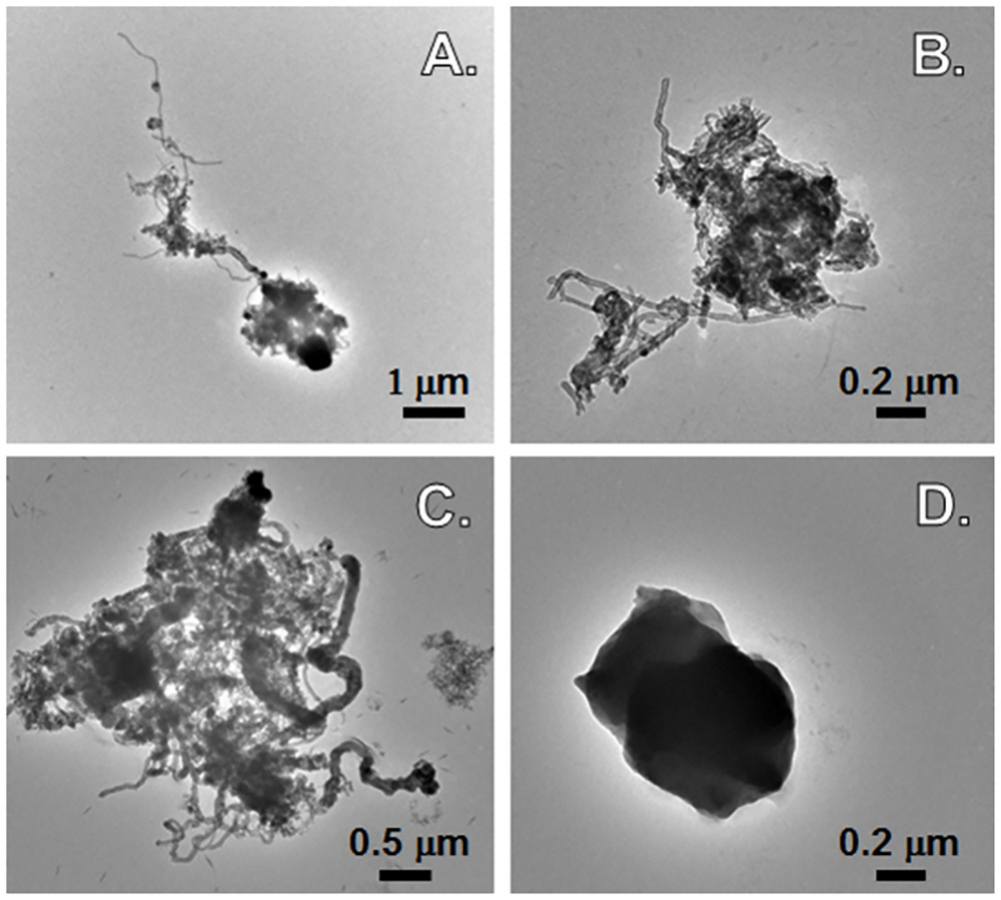
Transmission electron micrographs of samples collected in MWCNT facilities. TEM images of filters collected from personal breathing zone sampling showing: A, more dispersed MWCNT particles; B-C, agglomerated structures from potentially exposed groups, and D, a media blank (stored in a clean room).

Representative TEM images of air samples from the MWCNT facility are shown in Fig 1. TEM analysis revealed several types of CNT particles in these samples. Most often, the particles were entangled, agglomerated structures measuring several μm crosswise (e.g., Fig 1A–1C). However, in some cases, curved fibers or bundles of long/slender CNT structures were associated with the agglomerates (e.g., Fig 1A). These findings are in line with previous studies that reported the presence of both tangled and more dispersed CNT structures in air samples from CNT manufacturing facilities [26, 45, 46].

The CNT structure counts for samples collected within the facility are reported in S2 Table. The table reports the number of structures counted per number of grid openings (e.g., Grid 1, mechanical grinding: 16 structures dispersed over 14 grid openings), the total number of structures per square millimeter of filter area (CNT/mm^2^), and the longest measured crosswise dimension for each structure. For each structure, two additional crosswise lengths were measured and averaged to give a rough estimate of the minor axis, for particles with oblate shapes, or a rough diameter for roughly spherical particles. These averages are not reported, but in most cases, the ratio of the maximum cross-wise length to the minor axis (average) was 4:1 or less, averaging about 1.5:1.

**Fig 2:**
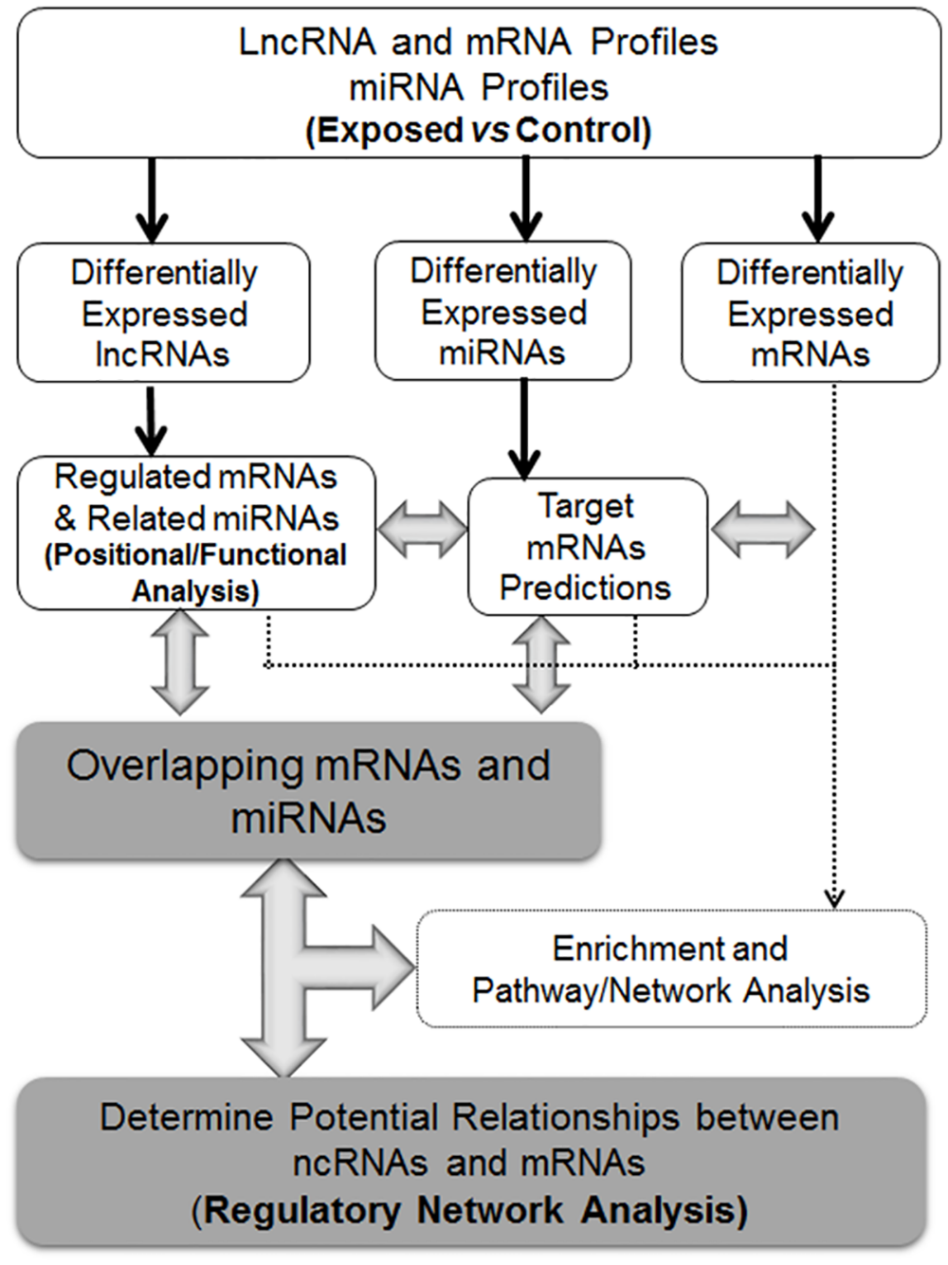
An overall schema summarizing the integrated bioinformatics analysis of differentially expressed ncRNAs and mRNAs to determine their potential relationships and interactions in MWCNT exposed and non-exposed workers.

The structures found were classified as two main types, ‘matrix’ or ‘cluster’ (S1 Fig). Matrix structures appear as particles with dark regions and associated CNTs (S1A and S1B Fig), and clusters are visible as mainly loose bundles of CNTs (S1C Fig). The most common structure was the matrix type, but clusters also were present. Based on the EDXA spectra, matrix particles contained several elements, while CNT clusters contained mainly nickel and lesser amounts of cobalt. Other types of structures (e.g., Fig 2D), without visible CNT, also were detected in all samples in varying amounts, possibly from air pollution or other sources. None of the clean room samples showed the presence of CNTs. However, TEM analyses of area and PBZ samples (Fig 1A–1D) collected at the MWCNT manufacturing facilities clearly indicate the presence of inhalable/respirable fractions of CNTs.

The EC results for personnel breathing zone samples from various processes that involve processing/handling MWCNT are listed in S3 Table. As with the TEM analyses, the EC results confirm the presence of airborne CNTs. Of the various processes investigated, PBZ samples collected in the harvesting and packaging areas exhibited the highest exposure levels (S3 Table), consistent with the TEM results. In these areas, the average inhalable EC concentration were found to be ~14.42 ± 3.8 μg/m^3^ (Table 1). Further, the calculated average EC concentrations, indicating the MWCNT concentrations to which the study subjects are potentially exposed on a daily basis, along with the approximate lung burdens estimated using MPPD (v.2.1) modeling software, are listed in Table 2. Taken together, these results clearly indicate potential MWCNT exposure at concentrations higher than the NIOSH recommended exposure limit (REL) of 1 μg/m^3^ for an 8 h time-weighted average of EC [47] (Table 2 and S3 Table).

**Table 1:** Elemental carbon (EC) concentrations found for personal breathing zone (PBZs) samples from workers performing different tasks.

Process	EC inhalable conc., μg/m^3^	EC respirable conc., μg/m^3^	%respirable fraction
Harvesting	29.6	6.11	20.65
Mechanical grinding	2.05	2.03	99.03
Packaging	25.3	2.65	10.48
Laboratory handling	0.71	0.54	76.06
**Mean ± Std. Error**	**14.42 ± 3.8**	**2.83 ± 0.6**	**-**

**Table 2:** Calculated inhalable (I) and respirable (R) elemental carbon (EC) concentrations of workforce and their approximate lung burdens estimated using MPPD (v.2.1) modelling software.

Group	Occupation (sample ID)	Gender	(I) TWA 8hr. EC conc., μg/m3	(R) TWA 8hr. EC conc., μg/m3	% respirable	Lung burden per year (μg)	Lung burden per day (μg)	Lung burden per day, mouse (ng)
**Exposed**	TB1	M	17.14	2.78	16.19	3949.92	15.8	7.74
	TB2	M	10.37	1.75	16.86	2389.25	9.56	4.69
	TB3	M	3.51	0.66	18.6	808.13	3.23	1.59
	TB4	M	3.51	0.66	18.6	808.13	3.23	1.59
	TB5	M	4.37	1.27	29.09	1005.84	4.02	1.97
	TB14	M	17.14	2.78	16.19	3949.92	15.8	7.75
	VB1	M	4.37	1.27	29.09	1005.84	4.02	1.97
	VB3	M	4.37	1.27	29.09	1005.84	4.02	1.97
**Non-Exposed**	TB6	M	-	-	-	-	-	-
	TB7	M	-	-	-	-	-	-
	TB9	M	-	-	-	-	-	-
	TB10	M	-	-	-	-	-	-
	TB12	M	-	-	-	-	-	-
	VB2	M	-	-	-	-	-	-
	VB4	M	-	-	-	-	-	-

The 8hr time-weighted average (TWA) EC concentrations for each occupation in the exposed group were calculated based on inhalable/respirable EC concentrations of PBZ samples (see Table 1) as well as time spent by the personnel in that particular unit/technological process during a working day.

### Differentially expressed ncRNAs and mRNAs in workers exposed to MWCNT

To profile differentially expressed non-coding and coding RNAs in MWCNT exposed workers, we performed analysis of miRNA, lncRNA and mRNA expression in the blood samples between MWCNT exposed and control groups. We found that a total of 977 lncRNAs were differentially expressed between MWCNT exposed and the non-exposed workforce groups based on statistical significance that passed our cutoff filtering (fold change ≥ ±1.5, P<0.05). Among them, 529 lncRNAs were upregulated and 448 lncRNAs were downregulated (S4 Table). Further, the statistical analysis of global shifts in mRNA expression revealed a total of 785 genes (fold change ≥ ±1.5, P<0.05) from MWCNT exposed group to show differential expression compared with samples from non-exposed group (S5 Table). Among these differentially expressed genes, 292 genes were up-regulated and 493 genes were down-regulated. In addition, out of a total of 11 differentially expressed miRNAs, 7 were found to be upregulated and 4 miRNAs to be downregulated in MWCNT exposed group. The comparison of miRNA and mRNA expression changes clearly indicate synergistic associations between ncRNAs and mRNAs. Most importantly, the changes in the expression profiles of ncRNAs and mRNAs were more prominent in three workers (sample IDs TB1, TB2, TB14), who are potentially exposed to high levels of MWCNTs (lung burden > 9 μg EC per day; Table 2). A total of 29 miRNAs (with 29**↑)**, 2404 lncRNAs (with 1571**↑** & 833**↓**) and 2655 mRNAs (with 1084**↑** & 1571**↓**) were differentially expressed between high MWCNT exposure and non-exposed group (S1 File).Overall, the mRNA/ncRNA expression changes in MWCNT-exposed workers were related to different occupations associated within a particular unit/technological process (Tables 1 and 2), rather than the number of years being employed in the manufacturing facility (~ 1–2 years with lung burden’s > 9μg EC per day for foreman/technical operators *versus* ~ 0.5–2 years for scientists/engineers with lung burden’s < 5μg EC per day).

### Association of Dysregulated mRNAs and miRNAs with Known Disease and Other Outcomes in Rodents

A detailed analysis of dysregulated miRNAs and mRNAs in the blood was performed using IPA to identify mRNAs and miRNAs associated with various pulmonary and systemic outcomes in rodents. A list of all differentially expressed mRNAs and miRNAs for each outcome in the overall and high MWCNT exposure groups is provided in Table 3. The dysregulated mRNAs and miRNAs upon exposure to MWCNTs were mostly associated with pulmonary inflammation and fibrosis, in agreement with many previous *in vivo* studies reporting inflammatory and fibrotic responses in rodents upon exposure to MWCNTs [14, 48–53]. Eleven mRNAs and three miRNAs in the overall MWCNT group, and sixty-two mRNAs and four miRNAs in the high dose group were associated with ‘inflammatory of the lung’ and/or ‘pulmonary fibrosis’. Several significantly changed mRNAs were also associated with systemic, cardiovascular and carcinogenic outcomes in the blood of workers exposed to MWCNTs. In the overall MWCNT exposure group, four mRNAs were associated with ‘vasodilation of arteries’ and seven mRNAs with ‘atherosclerotic lesions’. Similarly for the high dose group, a total of seventeen and eighteen mRNAs were associated with ‘atherosclerotic lesions’ and ‘vasodilation of arteries’. While in the overall exposure group significant mRNAs and miRNAs associated with carcinogenic and other outcomes considered were either absent or scarce, in the high dose MWCNT exposure group eight mRNAs—IL6, LTA, TREM2, HGF, VEGFA, CD44, STAT1, BRAF—were associated with ‘granuloma/formation of granuloma’; two mRNAs—OLR1, LGALS3 –were associated with ‘systemic inflammation’; seven mRNAs: TP63, TNK1, TP73, BRAF, XPA, HTAT1P2, NUDT1, and two mRNAs: EGFR, TNRC6B were associated with ‘pulmonary adenoma’ and ‘broncho-alveolar lung cancer’, respectively; nine mRNAs—PTGS2, PTN, Integrin, Jnk, S100A10, Cdk, FHIT, ATP Synthase, RARB—were associated with ‘formation of lung tumor’; three mRNAs—HRH1, ADORA1, LGALS3 –were associated with ‘goblet cell metaplasia’; and five mRNAs—IKBKE, RORA, GATA3, CDH13, TSLP—were with ‘goblet cell hyperplasia’. Importantly, many of these mRNAs had expression direction (up/down regulation) consistent with their known roles leading to increase in pulmonary and other disease outcomes (Table 3, highlighted in bold). For example, out of a total of sixty-six mRNAs and miRNAs, twenty-three mRNAs and a single miRNA (miR-21-5p) are known to increase pulmonary inflammation and/or fibrosis from IPA knowledge base and were in fact found to be significantly up-regulated in the blood in the high exposure group. Taken together, these results clearly indicate that mRNAs and miRNAs in the workers of high exposure group, rather than the overall MWCNT exposed group, are more reminiscent of biological responses upon exposure to engineered nanomaterials. It is well documented that exposures to engineered nanomaterials, in particular MWCNT, results in pulmonary inflammation, fibrosis, tumor promotion and cancer progression. Thus, the differentially expressed mRNAs, lncRNAs and miRNAs of only the high exposure group will be subjected to a detailed bioinformatics analysis, as summarized in Fig 2.

**Table 3:** mRNAs and miRNAs in the blood of MWCNT exposed workers associated with various pulmonary and systemic outcomes.

	MWCNT overall exposure group	MWCNT high exposure group
**Lung Inflammation and/or Fibrosis**	**mRNAs:** ↑EDNRA, ↑ACKR1, **↑TRPV4,** ↑LTA, **↑CSF2,** ↓P2RY12, ↓CXCL3, **↓CCL3L3,** ↓FCGRT, ↓TUBB, ↓RORC	**mRNAs:** ↑EDNRA, ↑PTGIR, **↑PTGS2, ↑IL6, ↑SPHK1, ↑FGFR1, ↑RETNLB, ↑PLG, ↑CSF2, ↑VTN, ↑CXCL12, ↑EGFR,** ↑PPARD, **↑TNFRSF25,** ↑CHRNG, ↑ACKR1, ↑FCGR2B, **↑CCR5, ↑TNSF4, ↑HRH1,** ↑ST3GAL3, ↑HMGCR, ↑IMPDH1, **↑CDH13,** ↑IKBKE, ↑LDLR, **↑BSG,** ↑CALCA, ↑MARK2, **↑RORA,** ↑LTA, ↑TFPI, ↑LIPA, **↑VEGFA, ↑TRPV4, ↑GATA3, ↑MAPK3,** ↑POMC, ↑CD276, ↓CD44, ↓STAT1, ↓GSS, ↓HLA-DRA, ↓IL11RA, ↓RLN2/3, ↓CD3D, ↓CD46, **↓TSLP,** ↓FGFR3, ↓LGALS3, ↓LIF, **↑TLN1,** ↓CHRNE, ↓SERPINB1, ↓ANXA1, ↓ADORA1, ↓IMPDH2, ↓PRDX, ↓E2F2, ↓CASP1, ↓CYLD, ↓NRTN, ↓C3AR1
	**miRNAs:** ↑hsa-miR-30a, ↑hsa-miR-140-3p, ↓hsa-miR-92a	**miRNAs:** ↑hsa-miR-140-3p, **↑hsa-miR-21-5p**, ↑hsa-miR-30a-3p, ↑hsa-miR-20a-5p, ↑hsa-miR-27b-3p
**Granuloma**	**mRNAs:** ↑LTA	**mRNAs:** ↑IL6, ↑LTA, ↑TREM2, **↑HGF, ↑VEGFA,** ↓CD44, ↓STAT1, ↓BRAF
**Immunosuppression**	—	**mRNAs: ↑TNFSF4,** ↓Pka
**Bronchoalveloar adenoma**	**mRNAs:** ↓XPA	**mRNAs:** ↑TP63,↑TNK1, ↓TP73, ↓BRAF, ↓XPA, ↓HTAT1P2, ↓NUDT1
**Bronchoalveolar adenoarcinoma**	**mRNAs:** ↑TTR	**mRNAs:** ↑EGFR, ↑TNRC6B
**Formation of Lung tumors**	**mRNAs:** ↑Integrin	**mRNAs: ↑PTGS2, ↑PTN, ↑Integrin,** ↑Jnk, ↓S100A10, ↓Cdk, ↓FHIT, ↓ATP Synthase, ↓RARB
**Lung hyperplasisa**	—	—
**Goblet cell metaplasia**	—	**mRNAs: ↑HRH1,** ↓ADORA1, ↓LGALS3
**Goblet cell hyperplasia**	—	**mRNAs:** ↑IKBKE, **↑RORA, ↑GATA3, ↑CDH13,** ↓TSLP
**Systemic Inflammation**	—	mRNAs: **↑OLR1,** ↓LGALS3
**Atherosclerotic lesions**	**mRNAs: ↑LTA, ↑VCAM1,** ↑GSTT1, **↑CSF2,** ↑BMP7, ↓Mmp, ↓CCL3L3	**mRNAs:** ↑GSTT1, **↑LTA,** ↑GPX8, ↑FUT4, ↑CCR5, **↑PLG,** ↑MMP19, ↑BMP7, **↑VEGFA, ↑CSF2,** ↑LDLR, **↑TNFSF4,** ↓FUT7, ↓CXCR1, ↓CD44, ↓CCL3L3, **↓FABP4,** ↓HMOX2, **↓CD3, ↓GRK4, ↓MIF, ↓LDL**
**Vasodilation of Arteries**	**mRNAs: ↑NOX1, ↓GUCY1A3,** ↓PDGFB, **↓RLN2**	**mRNAs:** ↑VEGFA, ↑MFAP5, **↑NOX1, ↑PIK3R1/R3, ↑PLG,** ↑LDLR, ↑CALCA, ↑ERK, ↑PLCD3, **↓HMOX2, ↓GUCY1A3,** ↓PDGFB, **↓SOD1, ↓RLN2/3, ↓PLCL2,** ↓PTPN1, **↓CELA1**

Significantly up- or down-regulated (FC >± 1.5, p<0.05) mRNAs/miRNAs of the overall and high MWCNT exposure groups versus non-exposed workers associated with each outcome were derived using IPA. miRNAs/mRNAs that are known to increase the disease/other outcomes considered are highlighted in bold.

### Identification and Analysis of lncRNA-mRNA interactions and miRNA-mRNA target pairs in workers exposed to MWCNT

As ncRNAs are known to participate in transcriptional, epigenetic, and/or post-transcriptional regulation of gene expression, we predicted and constructed the expression profiles of the target mRNAs using differentially expressed miRNAs and lncRNAs. Detailed analysis of dysregulated lncRNAs and their 850 nearby coding genes identified a total of 133 differentially expressed lncRNAs with differentially expressed nearby coding genes (distance <300 kb) in MWCNT-exposed workers (S1 & S4 Files). Out of these 133 pairs of differentially expressed lncRNAs and nearby coding genes, 68 pairs were found to be consistent with the direction of lncRNA expression changes and their sense/antisense relationship (S4 File). The expected expression of some nearby coding genes of dysregulated lncRNAs was inconsistent with the mRNA expression changes (S4 File). Further, the analysis of genes targeted by miRNAs, performed using target prediction algorithms, revealed a total of 5442 mRNAs to be targeted by a total of 19 miRNAs differentially expressed in the high MWCNT exposure group. Following this, we integrated the predicted potential miRNA based mRNA targets with the differentially expressed mRNAs profiles in each case (fold change ≥1.5, P<0.05). A total of 115 matched miRNA-mRNA pairs for 5 differentially expressed miRNAs and 106 differentially expressed mRNAs were observed for the MWCNT exposed group; and a total of 1232 matched miRNA-mRNA pairs for 18 differentially expressed miRNAs and 670 differentially expressed mRNAs for the high dose MWCNT exposed group. The list of top 5 up- and down-regulated genes in each case along with their known disease associations from IPA knowledge base is provided in S6 Table. Compared to the overall MWCNT exposed group, the top ranked dysregulated genes of the highdose group had a significant overlap with known pathological outcomes (e.g., adenocarcinoma, adenoma, fibrosis and tumorigenesis) in rodents upon pulmonary exposure to MWCNTs [2, 14, 48, 49, 53–56].

### IPA Core Analysis of Differentially Expressed mRNAs, miRNAs and miRNA-mRNA target pairs in the whole blood of workers exposed to MWCNTs

Table 4 presents the associated categories of diseases and disorders, molecular and cellular functions as well as physiological system development and functions in the blood of workers exposed to MWCNT.

**Table 4:** Functional analysis of differentially expressed mRNAs, miRNAs and miRNA-mRNA target pairs in the whole blood of workers exposed to MWCNTs.

mRNA	miRNAs	miRNA-mRNA targets
**Top Five Diseases and Disorders (# of genes / miRNA)**		
1. Cancer (1932)	1. Cancer (19)	1. Neurological Disease (132)
2. Organismal Injury and Abnormalities (1965)	2. Gastrointestinal Disease (17)	2. Psycological Disorders (63)
3. Infectious Diseases (296)	3. Organismal Injury and Abnormalities (19)	3. Skeletal & Muscular Disorders (79)
4. Gastrointestinal Disease (1318)	4. Respiratory Disease (14)	4. Hereditary Disorder (85)
5. Hematological Disease (390)	5. Reproductive System Disease (12)	5. Tumor Morphology (7)
**Top Five Cellular and Molecular Functions (# of genes / miRNA)**		
1. Cell Death and Survival (636)	1. Cellular Movement (10)	1. RNA Post-transcriptional Modification (27)
2. Protein Synthesis (230)	2. Cell Development (13)	2. Cellular Development (60)
3. Gene Expression (413)	3. Cellular Growth and Proliferation (13)	3. Cellular Growth and Proliferation (57)
4. Cellular Growth and Proliferation (644)	4. Cell Death and Survival (8)	4. Cell to Cell Signaling and Interaction (45)
5. RNA Post-transcriptional Modification (73)	5. Cell Morphology (4)	5. Nucleic Acid Metabolism (27)
**Top Five Physiological System Development and Functions (# of genes / miRNA)**		
1. Organismal Survival (442)	1. Organismal Development (6)	1. Organismal Survival (138)
2. Organismal Development (404)	2. Digestive System Development and Function (2)	2. Tissue Development (53)
3. Tissue Morphology (445)	3. Hepatic System Development and Function (2)	3. Auditory and Vestibular System Development and Function (8)
4. Hematological System Development and Function (317)	4. Organ Development (3)	4. Hematological System Development and Function (52)
5. Immune Cell Trafficking (161)	5. Hematopoiesis (3)	5. Immune Cell Trafficking (16)

Significantly up- or down-regulated (FC >± 1.5, p<0.05) mRNAs/miRNAs of the high MWCNT exposure group versus non-exposed workers associated with ‘disease and disorder’, ‘cellular and molecular functions’, and ‘physiological system development and function’ were determined using IPA.

#### Functional Analysis

IPA core analysis based on changes in both mRNA, miRNA and their mRNA target expression profiles reflect similar outcomes upon exposure to MWCNT. The diseases and disorders “Cancer”, “Gastrointestinal Disease”, and “Organismal Injury and Abnormalities” were commonly predicted to be among the top 5 diseases perturbed by differentially expressed mRNAs, miRNAs and miRNA-mRNA target pairs (Table 4). Additionally, the functional analysis also indicated significant association of genes and miRNAs with cancer that functioned primarily in cell death and survival and cellular growth and proliferation, upon exposure to MWCNTs in humans. In addition to their similarities in the disease/disorder and molecular/cellular functions categories, the dysregulation of both mRNA and miRNA expression profiles also indicated similarities within each subcategory. For example, in the case of the top-ranked disease/disorder category “Cancer”, the subcategories lymphoid cancer, hematologic cancer, gastrointestinal tract cancer, digestive system cancer, colorectal cancer and non-small cell lung cancer (NSCLC) were common. Most importantly, the predicted involvement of disease/disorder category, NSCLC is in line with previous studies, which reported significant DE of several genes markers of NSCLC and lung carcinogenesis upon exposure to MWCNT in rodents. Additionally, IPA analysis also identified “Respiratory Disease” as common to both changes in mRNA and miRNA expression and one of the top 5 most significant disease and disorders based on miRNA analysis (Table 4). Further analysis of “Respiratory Disease” category indicated similar subcategorical disease outcomes related to NSCLC, and lung carcinoma. This analysis points out a synergistic association between both miRNAs and mRNAs in response to MWCNT exposures in humans. Moreover, changes in miRNA expression profiles also identified (naso-)pharyngeal carcinoma, squamous cell cancer, respiratory system tumor, idiopathic pulmonary fibrosis, and lung cancer as the most significantly perturbed “Respiratory disease” outcomes upon exposure to MWCNT in humans. Overall, the IPA functional analysis of dysregulated mRNAs, miRNAs and its gene targets, clearly suggests the potential of MWCNT in triggering carcinogenic and other pulmonary disease outcomes in humans, similar to those seen previously in rodent models [2, 14, 48, 49, 53–56].

#### Gene Signaling Network Analysis

**Fig 3:**
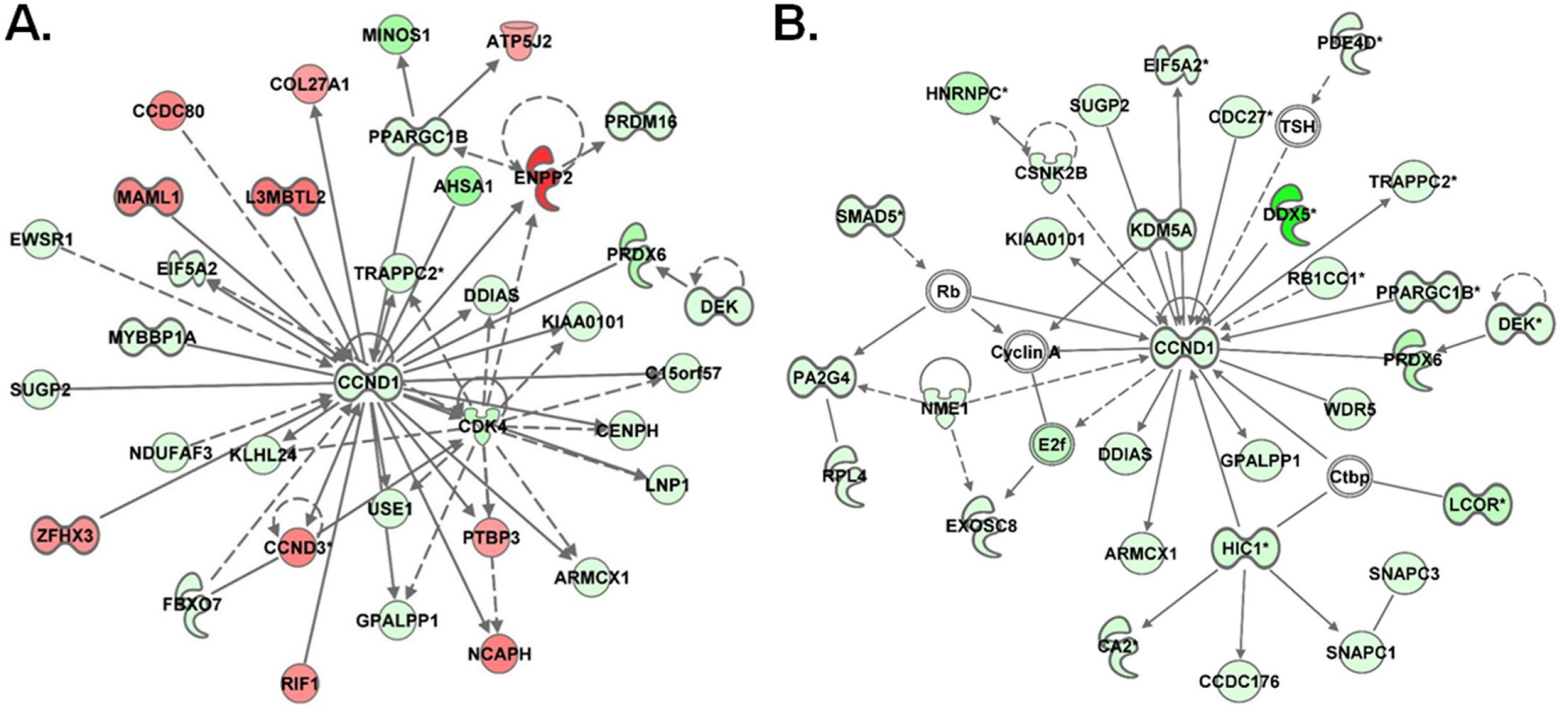
Highest scoring gene molecular networks of mRNAs and miRNA-mRNA target pairs in MWCNT high exposure group. Gene signaling network analysis by IPA of differentially expressed (A) mRNAs and (B) miRNA-mRNA target pairs in high MWCNT exposure group. The datasets either containing all differentially expressed genes or genes having consistent expression direction based on differentially expressed miRNAs were uploaded into IPA then analyzed with the cutoff criteria of ≥ 1.5 fold change and a p-value < 0.05. Only the network is presented for each group. Intensity of the red (upregulated) or green (down regulated) color of the nodes in the graph indicates level of gene expression.

The IPA top-ranked gene signaling networks (GSNs) were evaluated to identify potential networks and mechanisms perturbed based on changes in the expression profiles of mRNAs and miRNA-mRNA target pairs in the whole blood upon exposure to MWCNTs. The top 10 gene networks predicted using IPA, along with significance scores, and number of focus molecules are listed in Table 5. The top-ranked signaling network based on miRNA-mRNA targets was “Cellular Growth and Proliferation, Gene Expression, Cell Cycle” (score: 39) and on mRNAs was “Cell Cycle, Cell-To-Cell Signaling and Interaction, Connective Tissue Development and Function (score: 33)” (Table 5). Despite differences in their associated disease and functions, top predicted GSNs of mRNAs and miRNA-mRNA targets upon MWCNT exposure in the whole blood predicted the involvement of oncogene Cyclin D1 (CCND1) as the hub gene. CCND1 encodes for an important cell cycle regulatory protein (Fig 3). A total of 11 genes (CCND1, EIF5A2, ARMCX1, TRAPPC2, DDIAS, KIAA0101, DEK, PRDX6, PPARGC1B, SUGP2, GPALPP1), out of the 35 focus genes were predicted to be common to the top-ranked GSNs—network 1 –based on both mRNAs and miRNA-mRNA target pairs (Fig 3). The biofunctions associated with genes downstream of CCND1 after MWCNT exposure were those with functional roles in arrest in G1 phase, cell cycle progression, proliferation, G1/S phase transition and size/morphology of cells and included cyclins (CCND1,CCND3), cyclin-dependent kinase 4 (CDK4), DNA damage-induced apoptosis suppressor (DDIAS), DEK proto-oncogene (DEK), peroxisome proliferator-activated receptor gamma (PPARGC1B) and others (Fig 3A). Similarly for miRNA-mRNA based target genes (Fig 3B), biofunctions associated with the top GSN network were centered on expression/transcription of RNA, apoptosis, proliferation and differentiation of cells, G1 phase, G1/S phase transition, arrest in G1 phase, delay in G1/S phase, cell cycle progression, cell division, oxidative stress response and included CCND1, cell division cycle genes (CDC27), DDIAS, DEAD (Asp-Glu-Ala-Asp) box helicase 5 (DDX5), hypermethylated in cancer 1 (HIC1), lysin specific demethylase A (KDM5A), proliferation associated 2G4 (PA2G4), DEK Proto-oncogene (DEK), peroxiredoxin 6 (PRDX6) and several others. Most importantly, CCND1, the hub gene common to the two top most significant GSNs, is known to play major role in cell cycle: G1/S checkpoint regulation, p53 signaling, aryl hydrocarbon receptor signaling, molecular mechanisms of cancer, (non-)small cell lung cancer signaling and others (Fig 3). Interestingly, the up regulation of MAML1 was implicated in regulation of epithelial-mesenchymal transition pathway and Notch signaling. Many genes common to both GSNs, CCND1, DDIAS, KIA0101, EIF5A2, PPARGC1B, PRDX6, DEK, were mostly implicated in Cell Cycle, Cellular Development/Movement/Growth and Proliferation, Inflammatory Response, Immune Cell Trafficking and Free radical Scavenging. The top 10 most significant GSNs generated based on mRNA and miRNA-mRNA target expression profiles in the blood of workers exposed to MWCNTs, along with significance scores, and respective hub genes are listed in Supplemental material, S2 File.

**Table 5:** Gene Signaling networks of differentially expressed mRNAs and miRNA-mRNA target pairs in the whole blood of workers exposed to MWCNTs.

Rank	mRNA	miRNA-mRNA targets
Top Ten Predicted Associated / Related Network Functions (Prediction Score / # of Focus Molecules)		
1	Cell Cycle, Cell-To-Cell Signaling and Interaction, Connective Tissue Development and Function (33/35)	Cellular Growth and Proliferation, Gene Expression, Cell Cycle (39/30)
2	Cell-To-Cell Signaling and Interaction, Carbohydrate Metabolism, Developmental Disorder (29/33)	RNA Post-Transcriptional Modification, Cell Death and Survival, Cellular Compromise (33/27)
3	Connective Tissue Development and Function, Hematological System Development and Function, Hematopoiesis (29/33)	Cell Death and Survival, Connective Tissue Disorders, Dermatological Diseases and Conditions (29/25)
4	Developmental Disorder, Hematological Disease, Hereditary Disorder (29/33)	Cellular Function and Maintenance, Cell Morphology, Infectious Diseases (29/25)
5	Cell-To-Cell Signaling and Interaction, Cellular Movement, Hematological System Development and Function (29/33)	Cell-To-Cell Signaling and Interaction, Drug Metabolism, Small Molecule Biochemistry (27/24)
6	Cell Cycle, DNA Replication, Recombination, and Repair, Cancer (27/32)	Cell-To-Cell Signaling and Interaction, Cell Morphology, Reproductive System Development and Function (26/23)
7	Antimicrobial Response, Inflammatory Response, Cellular Function and Maintenance (27/32)	Cardiovascular System Development and Function, Cell-To-Cell Signaling and Interaction, Renal and Urological System Development and Function (26/23)
8	Skeletal and Muscular System Development and Function, Connective Tissue Development and Function, Tissue Development (27/32)	Cell Cycle, Inflammatory Disease, Ophthalmic Disease (26/23)
9	Gene Expression, Connective Tissue Development and Function, Organismal Development (27/32)	Cell Cycle, Cellular Development, Hematological System Development and Function (22/21)
10	Small Molecule Biochemistry, RNA Post-Transcriptional Modification, Drug Metabolism (27/32)	Cancer, Organismal Injury and Abnormalities, Embryonic Development (19/19)

### Pathway enrichment analysis of mRNAs and miRNA-mRNA targets in the whole blood samples of workers exposed to MWCNTs

#### KEGG pathways enriched by dysregulated mRNAs

**Fig 4:**
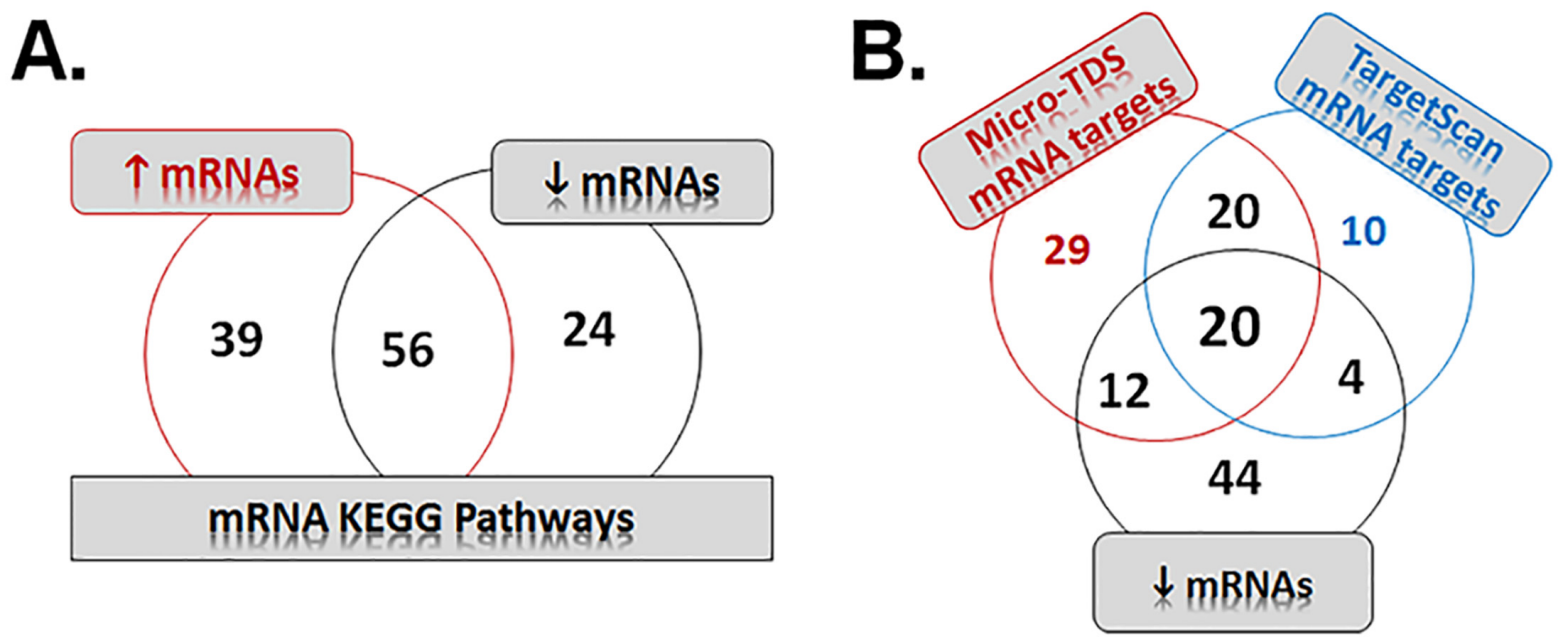
KEGG pathway enrichment analysis of dysregulated genes and miRNA-mRNA target pairs in the MWCNT-exposed workers. Venn diagrams showing common and unique enriched pathways of (A) up- and down-regulated mRNAs KEGG pathways, and (D) down-regulated mRNAs and miRNA-mRNAs targets identified using micro-TDS and TargetScan algorithms of DIANA-miRPath.

Signaling pathways analysis of differentially up and down regulated genes, using DAVID [41, 57], predicted the involvement of 95 and 80 pathways (p<0.05), respectively, in the blood of MWCNT high exposure group. A total of 56 pathways, related to cancer (e.g., pathways in cancer, small- and non-small cell lung cancer), cellular processes (e.g., apoptosis, focal adhesion, regulation of actin cytoskeleton, endocytosis, axon guidance, cell cycle, gap- and tight-junction signaling), stress and injury (e.g., cytokine-cytokine- and ECM-receptor interactions, cell adhesion molecules, MAPK, PPAR, Jak-STAT, mTOR, Wnt, Calcium signaling), immune system (e.g., Hematopoietic cell lineage, Natural killer cell mediated cytotoxicity, RIG-I-like receptor, Toll-like receptor, chemokine) and circulatory system/cardiovascular disease (e.g, dilated cardiomyopathy, vascular smooth muscle and cardiac muscle contraction), were commonly enriched by both up and down regulated mRNAs (Fig 4A, S3 File). Many of these signaling pathways, including apoptosis, cell cycle regulation and mitogen activated protein kinase (MAPK) signaling and disease pathways related to cancer/systemic repsonses, were previously reported to be activated upon exposure to CNTs [58–65]. In addition to these commonly identified signaling mechanisms, pathways related to nervous system (e.g., long-term potentiation and depression), neurodegenerative diseases (e.g., Huntington’s, Parkinson’s, Alzheimer’s) and processing genetic information including those involved in transcription/translation/degradation (e.g., ribosome, proteasome, RNA degradation, ubiquitin mediated proteolysis) and replication/repair (e.g., Nucleotide excision repair, recombination), were found to be uniquely enriched by down-regulated genes upon MWCNT exposure. The pathways related to signal transduction pathways (e.g, ErbB, VEGF, TGFβ), immune system (e.g., Fc gamma R-mediated phagocytosis, Leukocyte transendothelial migration, complement and coagulation cascades, T- and B-cell, NOD-like receptor signaling) and endocrine system/metabolic diseases (e.g., Adipocytokine signaling, type I and type-II diabetes), were uniquely represented by up-regulated genes. Importantly, the dysregulation of genes that participate in signaling pathways that are common to both up/down regulated genes clearly highlights and support various adverse outcomes found previously in rodents and further suggests the potential of MWCNTs to trigger similar responses in humans.

#### KEGG pathways enriched by dysregulated miRNA-mRNA target pairs

To identify the biological pathways enriched by the mRNA targets of the miRNAs upregulated in workers exposed to MWCNTs, we performed an *in silico* based analysis of the putative interactions between the mRNAs targets and the most common KEGG pathways using DIANA-miRPath [44]. With the use of a threshold P value of 0.001, a total of 40 significantly enriched pathways were commonly enriched based on both TargetScan and micro-TDS mRNA target prediction algorithms (Fig 4b, S3 File). Many of these pathways were previously described upon exposure to CNTs, including MAPK, P53, PI3-Akt, transforming growth factor-beta (TGFβ), and Wnt signaling pathways, as well as regulation of cell cycle, apoptosis, focal adhesion, and pathways/transcription misregulation in cancer [47, 58–61, 66]. Further comparison of miRNA-mRNA enriched signaling pathways with those enriched by down-regulated mRNAs, identified overlapping pathways related to Apoptosis (e.g., apoptosis, JAK/Stat, Toll-like receptor signaling), Cellular growth/proliferation/development (e.g., Jak/STAT, Gap junction, mTOR), and disease specific pathways (e.g., P53, Pathways in cancer, NSCLC, SCLC, Dilated cardiomyopathy). Interestingly, a significant number of pathways were also found to be significantly enriched in organismal systems including endocrine (e.g., GnRH-, Insulin signaling) and nervous (e.g., neurotrophin signaling, long-term potentiation) systems. In fact, filtering for miRNA-mRNA targets with those having consistent down-regulation in the mRNA expression profiles, indicated enrichment of overlapping pathways mostly related to cancer (e.g., pathways in cancer, NSCLC) and cardiovascular/circulatory system effects (e.g., cardiac muscle contraction, vascular smooth muscle contraction) as well as cell cycle/movement (Table 6).

**Table 6:** KEGG signaling pathway enrichment of miRNA-mRNA targets in the whole blood of workers exposed to MWCNTs.

Rank	KEGG pathway	p-value	#genes	#miRNAs
1	Prion diseases	1.71E-46	2	2
2	Synthesis and degradation of ketone bodies	1.87E-08	3	3
**3**	**Butanoate metabolism**	**5.25E-07**	**4**	**3**
**4**	**Valine, leucine and isoleucine degradation**	**2.88E-06**	**4**	**2**
**5**	**Pathways in cancer**	**6.62E-06**	**8**	**6**
6	Thyroid cancer	4.52E-05	3	4
**7**	**Non-small cell lung cancer**	**4.77E-05**	**3**	**3**
8	Folate biosynthesis	0.00027464	1	1
**9**	**Fatty acid metabolism**	**0.00030932**	**2**	**2**
**10**	**Bladder cancer**	**0.0007482**	**3**	**3**
**11**	**Prostate cancer**	**0.00215104**	**4**	**3**
**12**	**Colorectal cancer**	**0.002305**	**3**	**3**
**13**	**Insulin signaling pathway**	**0.00251161**	**5**	**3**
14	Endometrial cancer	0.00267391	3	3
**15**	**Melanoma**	**0.00312374**	**3**	**3**
**16**	**Cell cycle**	**0.00314939**	**5**	**3**
17	Terpenoid backbone biosynthesis	0.00321421	2	2
**18**	**Chronic myeloid leukemia**	**0.00414792**	**3**	**4**
**19**	**Focal adhesion**	**0.00546325**	**6**	**6**
**20**	**Small cell lung cancer**	**0.00576343**	**3**	**2**
21	Herpes simplex infection	0.00589366	6	4
**22**	**Long-term potentiation**	**0.00806107**	**3**	**2**
**23**	**Oocyte meiosis**	**0.00987416**	**4**	**2**
**25**	**Pancreatic cancer**	**0.02183958**	**3**	**3**
**24**	**Progesterone-mediated oocyte maturation**	**0.02183958**	**3**	**2**
26	Fatty acid elongation	0.02315225	1	1
**27**	**Vascular smooth muscle contraction**	**0.03280908**	**3**	**2**
28	Vasopressin-regulated water reabsorption	0.03827082	2	1
**31**	**Cardiac muscle contraction**	**0.04415137**	**2**	**2**
**29**	**Vibrio cholerae infection**	**0.04572276**	**2**	**2**
30	Non-homologous end-joining	0.0464634	1	1

The KEGG pathway enrichment analysis was performed using DIANA-mirPath [44]. The mRNA targets of significantly up-regulated (FC > 1.5, p<0.05) miRNAs of the high MWCNT exposure group versus non-exposed workers were predicted using TargetScan algorithm and the resulting mRNA target list was further filtered for those with significant down-regulation (FC > 1.5, p<0.05) in the mRNA expression profiles obtained from same samples. The enriched pathways overlapping with those predicted based on all down-regulated mRNAs, using DAVID, are highlighted in bold.

Overall the pathway analysis of mRNAs and miRNA-mRNA targets in the blood of workers exposed to MWCNTs suggests the potential of MWCNTs in triggering carcinogenic and cardiovascular outcomes as well as highlights the synergistic role of mRNAs and miRNAs in driving such responses.

### Regulatory Network Generation and Analysis of ncRNA-mRNA co-expression and interactions in the whole blood of workers exposed to MWCNTs

**Fig 5:**
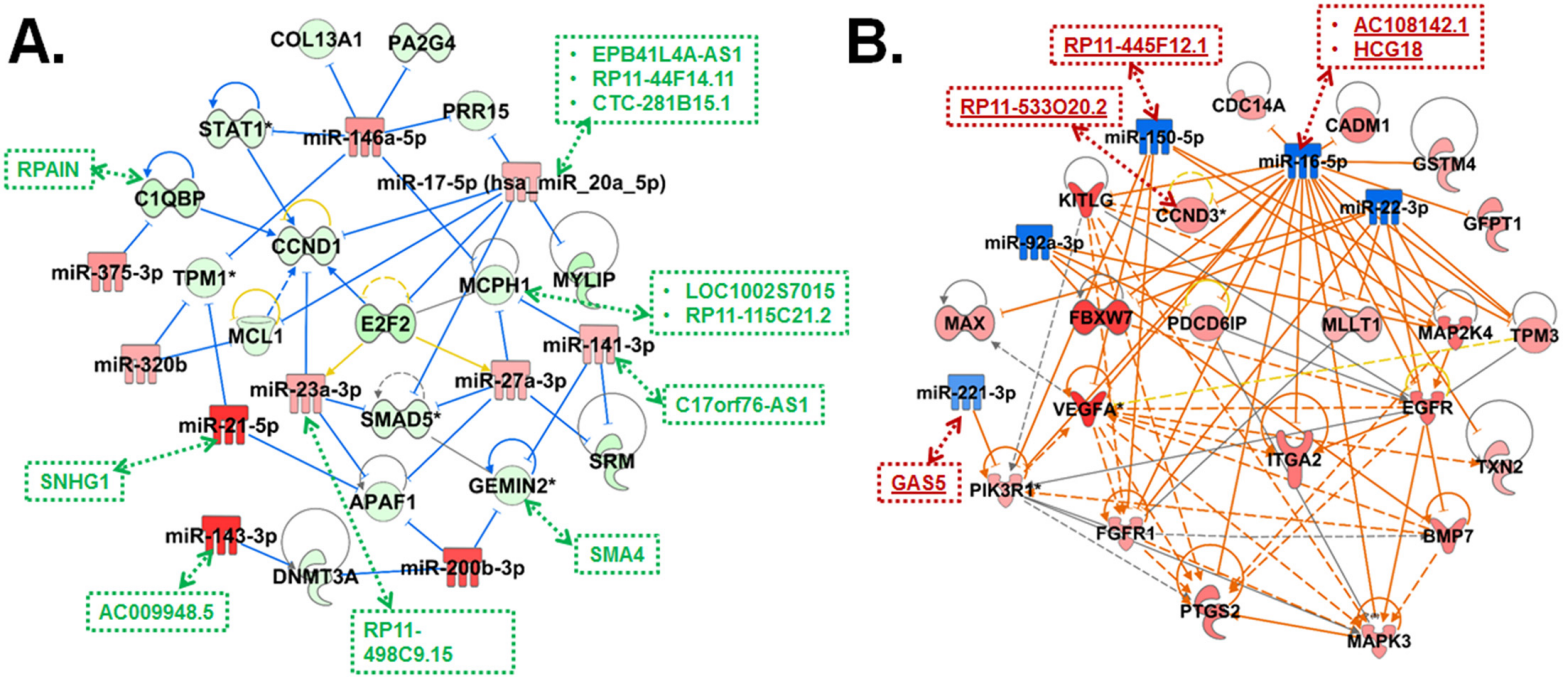
The regulatory network of ncRNAs and mRNAs in workers exposed to MWCNTs. The regulatory networks of significantly dysregulated lncRNAs and mRNAs (p-value<0.05, fold-change>±1.5) having positional and/or functional relationship with (A) up-regulated miRNAs (p<0.05, fold-change>1.5) and (B) down-regulated miRNAs (p>0.05, fold-change>-1.5), generated using IPA. The lncRNAs, represented using red (↓) and green (↑) rectangles and dotted lines, with consistent co-expression as mRNAs and/or containing miRNA target binding sites from starBase v2.0 were manually added to the network.

To identify and understand the synergistic interactions and overall patterns in the dysregulation of ncRNAs and mRNAs, a comprehensive analysis of their potential relationships was performed to generate a regulatory network by integrating the co-expression changes, interactions and positional details of lncRNAs, mRNAs and miRNAs. As a first step, all experimentally validated mRNA target pairs of significantly differentially expressed miRNAs (p<0.05, fold-change>±1.5) were selected and further filtered for their consistent differential expression in mRNA profiles. Following this, the direct and indirect interactions as well as regulatory relationships between these biological molecules were identified using IPA and was represented as a network. Some miRNAs, such as miR-20a-5p, a member of miR-17-92 cluster, and miR-146a-5p, were located in the central positions with multiple target mRNAs. Most of the mRNAs (e.g., CCND1, SMAD5) also showed consistent dysregulation patterns based on their up and/or downstream targets (Fig 5). The information corresponding to the positional relationships of mRNA/miRNA and lncRNAs were further incorporated into the regulatory network, to identify the role of lncRNAs in further regulating this network. Often these mRNA-lncRNA pairs had sense/antisense relationships, indicating their consistent expression (S4 File). The overall regulatory network indicated that the signaling mechanism triggered by mRNAs was prone to be downregulated in MWCNT-exposed workers.

None of the dysregulated miRNAs identified were significantly downregulated in MWCNT-exposed workers. However, the functional analysis of all dysregulated mRNAs indicated activation of mechanisms and pathways involving up-regulated genes (Figs 3A and 4A; S3 File), with key roles in several disease outcomes (e.g., cancer, respiratory diseases, fibrosis). Moreover a number of upregulated mRNAs had consistent co-expression and positional relationship to lncRNAs (S4 File), suggesting their regulatory roles in MWCNT-induced biological responses. Thus, down regulated miRNAs identified in high MWCNT exposure group, albeit with no statistical significance, were considered to generate a regulatory network (as described above) that accounts for and incorporates signaling mechanisms associated with the up-regulated mRNAs. The hsa-miR-16-5p was located in the central position with most of the target mRNAs. The lncRNA RP11-533O20.2 has its expression consistent with one of its nearby coding gene, CCND3. The expression of some downregulated miRNAs including hsa-miR-16-5p, despite their low statistical significance, were consistent with and also showed coordinated changes with respect to significantly dysregulated lncRNAs (Fig 5B). Interestingly, the generated regulatory networks of ncRNAs and mRNAs indicated that biological functions and diseases represented by these molecules were mostly associated with cellular growth/development/proliferation, cell death/apoptosis and cancer. Overall the regulatory network analysis suggests that the dysregulation of biological pathways and mechanisms in MWCNT-exposed workers is mediated by coordinated changes at the genetic and epigenetic stages of the transcriptome.

### Validation of aberrantly expressed genes corresponding to mRNAs and miRNA-mRNA target pairs by qRT-PCR

The differences in gene expression found by microarray analyses were validated by using quantitative real time RT-PCR (qRT-PCR). We chose a total of six genes (CCND1, CCND3, MUC1, E2F2, TGFβ1 and SOD1) with ≥ ± 1.5 fold dysregulation in the whole blood of MWCNT-exposed workers. Some of the selected genes (e.g., CCND1, E2F2, CCND3) are known targets of the miR-16-5p or other dysregulated miRNAs belonging to the oncogenic miR-17-92 family in MWCNT-exposed workers. The expression of CCND3 and MUC1 was up-regulated and CCND1, E2F2 and SOD1 were down-regulated in RT-qPCR studies, in accordance with their differential expression in the same subjects exposed to MWCNTs (Table 7). Although some variation concerning the degree of regulation was observed, the overall data obtained with microarrays were substantially confirmed by qRT-PCR for almost all genes associated with cell cycle regulation and fibrotic responses, with the exception of TGFβ1(Table 7). The real-time PCR result of TGFβ1 showed an opposite trend in its regulation compared to its microarray expression changes. While TGFβ1 showed a negative regulation based on minimum detection limit values for two of the three exposed subjects and was not considered for initial analysis, several of its up-/down-stream regulators showed expression consistent with the up-regulation of TGFβ1. Surprisingly, the RT-qPCR result of TGFβ1 is in line with this. Of the five genes that showed the same trends of up- and down-regulation as the microarray data, four of them were statistically significant (p<0.05), supporting an overall consistency (Pearson r = 0.79, *P*<0.05) between the RT-qPCR results and the microarray data.

**Table 7:** Real time PCR validation of microarray expression data using select genes in the whole blood of workers exposed to MWCNTs.

Gene	Microarray Data FC (range)	RT-qPCR Data (range)
CCND1	-1.8 (-1.4 to -2.3)	-2* (-3.3 to -1.2)
CCND3	2.4 (2.8 to 2.0)	1.64* (1.1 to 2.4)
E2F2	-5.1 (-2.9 to -8.6)	-1.4 (-2.4 to 1.2)
MUC1	3.4 (4.9 to 1.9)	1.6* (1.1 to 2.2)
SOD1	-6.2 (-3.3 to -7.4)	-1.5* (-2.3 to 1.0)
TGFβ1	-1.4 (-1.3 to -1.5)	1.5* (-1.2 to 2.5)

Isolated mRNA was reverse-transcribed and subjected to RT-qPCR reaction with Roche Taqman probes. Data represent Mean fold change values along with their lower and upper limits of expression changes, n = 3.

*p < 0.05.

## Discussion

To date, there has been no report of definitive disease and/or potential biomarkers of early health effects in humans exposed to CNTs. The whole blood gene expression profiling, as surrogate to tissue, has become a powerful and an informative approach to investigate various disease states and identity biomarkers. This pilot study has revealed changes in the lncRNA, miRNA and mRNA expression profiles in the whole blood samples of MWCNT exposed humans and suggests the potential of MWCNTs in triggering carcinogenic, pulmonary and cardiovascular outcomes as well as highlights the synergistic role of mRNAs, miRNAs and lncRNAs in driving such responses. This study is the first to show a total of 977 lncRNAs, 785 mRNAs and 17 miRNAs were differentially expressed (fold change ≥±1.5, P<0.05) between MWCNT exposed and the non-exposed workforce groups. We found that these changes were much more prominent in a sub-set of three individuals (Table 2, Sample IDs: TB1, TB2 and TB14) that were associated with specific roles within an occupational setting (e.g., foreman, technical operator) where they were exposed to higher EC concentrations than other workers in the facility (Table 2).The changes in expression profiles revealed a total of 2404 lncRNAs, 2655 mRNAs and 29 miRNAs to be differentially expressed among these occupations compared to non-exposed workforce employed in the same facility. This suggests an occupation dependent potential exposure to high levels of MWCNTs (Table 1). This is in line with the detailed TEM and EC analysis of PBZ samples collected at the MWCNT manufacturing facilities that determined highest exposure levels in harvesting and packaging areas. While the TEM analysis clearly indicated the presence of inhalable/respirable fractions of CNTs, EC analysis suggested potential exposure to CNTs at concentrations higher than the NIOSH recommended exposure limit (REL) of 1 μg/m^3^ for an 8 h TWA (Table 2 and S3 Table).

Biological responses associated with CNTs exposure can vary by the type of CNTs (e.g, single-, double- and multi-walled; long and short, tangled or straight), pulmonary exposure methods employed (e.g, inhalation, intra-tracheal instillation, or pharyngeal aspiration), and the dose of CNTs investigated [14, 49–51, 67, 68]. Despite these variations, the pulmonary toxicity studies of CNT exposure in rodents conducted to date have reported consistent non-cancerous adverse pulmonary effects including inflammation, evidence of oxidative stress, epithelial-mesenchymal transition and fibrosis [14, 48, 49, 51, 67, 68]. This is also evident in our study where changes in mRNA and miRNA expression profiles in the whole blood of MWCNT exposed workers was associated with various pulmonary outcomes (Table 3). The number of dysregulated mRNAs and miRNAs associated with pulmonary inflammation and fibrosis were considerably more compared to other outcomes, in agreement with CNT-induced early events or responses reported by many previous *in vivo* studies. Several of these mRNAs, including cytokines (e.g., IL6, CSF2, CXCL2), growth factors (e.g., VEGFA, PDGFA), and miRNAs (e.g., hsa-mir-21-5p, has-mir-27b-3p) are all known to increase inflammation and/or fibrosis. A recent study that investigated miRNA/mRNA expression profiles also reported the overexpression of mir-27b-3p in the blood from mice exposed to MWCNT via Inhalation [54]. In fact, the up-regulation of mir-27b-3p was exclusive to the fibrosis category, confirmed pathologically in the lungs [54]. Previous *in vivo* studies using rodent models clearly revealed a profibrotic potential of MWCNTs triggered by TGFβ, one of the key profibrotic mediators in the pathogenesis of pulmonary fibrosis. While, no change related to expression levels of TGFβ was found in MWCNT exposed workers, a significant enrichment of the TGFβ signaling pathways overrepresented by the up-regulated genes and the dysregulation of several mediators either involved in its signaling (e.g., SP1, SMAD6, MAPK, BMP7) or up-/down-stream (e.g., IL6, PTGS2, EGFR, MEF2D, PDGFA, FN1) had expression consistent with predicted activation or up-regulation of TGFβ1 in workers exposed to MWCNTs. Moreover, many of these genes have been linked to the development of fibrosis. Several chronic inflammatory conditions including fibrosis is a key marker in asbestos-induced pathological responses. Several *in vivo* and *in vitro* studies suggest that CNTs elicit biological responses similar to those observed for asbestos fibers [15, 69]. Importantly, exposure to MWCNTs and asbestos fibers revealed similarities in the expression of various markers of mesothelioma and/or cancer [52, 70]. The expression levels of some of these exposure markers, in particular TGFβ1 and MUC1, were found to be significantly increased in the whole blood samples of MWCNT exposed workers (Table 7). The levels of these markers observed in our study are in line with a recent pilot cross-sectional study which reported elevated levels of MUC1 and TGFβ1 in the induced sputum or serum samples of MWCNT exposed workers [71]. The serum levels of MUC1 or Krebs von den Lungen-6 (KL-6) have been demonstrated to be a useful marker for the diagnosis and monitoring of patients with various interstitial lung diseases including pulmonary fibrosis[72–74]. Morover, a positive association between the serum levels of MUC1/ KL-6 and risk of lung cancer was also reported [75]. This is compatable with a fibrogenic and/or carcinogenic potential of MWCNTs in humans, similar to that of asbestos fibers [15, 52, 69].

In addition to the early non-cancerous pulmonary effects, some *in vitro* and *in vivo* studies have shown genotoxic or carcinogenic effects upon exposure to MWCNTs. Inhalation exposure to MWCNT has been linked with lung fibrosis that could lead to epithelial to mesenchymal transition and promotion of tumor formation/progression/metastasis. Several cell-adhesion molecules (e.g., ITGA2, VCAM1, and COL4A) and extracellular-matrix proteins (e.g., FN1, VTN) exhibited dysregulation upon exposure to MWCNTs in humans. Especially, the overexpression of some of these EMT related factors are known to further support tumorigenic responses. While the increased expression of VCAM1 promotes tumor invasion and metastasis [76], high levels of VTN and/or FN1 in circulation have been reported to promote tumor formation and metastasis [77–79]. Similarly, their overexpression in the blood samples of MWCNT exposed workers is also in complete agreement with rodent studies which reported the promotion of lung tumor/adenoma/adenocarcinoma formation, progression and metastatic upon treatment with either SWCNTs or MWCNTs [56, 66, 80, 81]. Further, the functional analysis of IPA pointed out a synergistic role of both miRNAs, and mRNAs in predicting various disease/disorder outcomes including “Cancer” and “Respitaroty Disease”. Consistent with previous studies, our results also identified various pulmonary adverse effects (e.g., NSCLC, respiratory system tumor, Idiopathic pulmonary fibrosis, and lung carcinoma) as subcategories within the Cancer and/or Respiratory Disease categories.

Based on the close relationship and association between inhalation of ambient ultra-fine particles and increased risk of cardiovascular diseases [82–84], it has been suggested that exposure to carbon-based nanomaterials including CNTs might have the potential to induce systemic and immune responses. Some of the top ranked “Physiological System Development and Functions” predicted (e.g., Hematological system, Immune cell trafficking) using IPA functional analysis in this study also supports this notion. Additionally, the enrichment of several KEGG signaling pathways related to circulatory/cardiovascular and immune system was also observed based on dysregulated blood gene expression profiles of workers exposed to MWCNTs. Previous studies on pulmonary exposure to CNTs have reported various systemic responses in animals, including an increase in inflammatory mediators in the blood, oxidative stress in distant organs/tissues (e.g., Aorta, Spleen), and myocardial infarction as well as increased plaque formation in an atherosclerotic mouse model [62, 64, 85–88]. Interestingly, several genes associated with “Atherosclerotic lesions” were also found to be dysregulated in MWCNT exposed workers. The expression of many of these genes (e.g., VCAM1, LTA, PLG, CSF2, VEGFA, and TNFSF4) was consistent with their role in exacerbating atherosclerotic lesions, further corroborating the ability of MWCNTs in triggering such systemic responses. Especially, the overexpression of VCAM1 in the overall exposure group, dominated by workers who spent less time in the facility or had low exposure to MWCNTs (Table 2), supports the fact that exposure to even low concentrations of MWCNTs can have significant systemic and immune responses, despite no or low pulmonary toxicity [85, 86]. Furthermore, pulmonary exposure to MWCNT was previously shown to reduce the ability of coronary arterioles to respond to dilators [65]. Similarly in this study, a significant decrease in the expression of many factors (e.g., SOD1, RLN2/3, HMOX2, CELA1, GUCY1A3, ADORA1, GNAS, CAMK2D), known to be associated with increased “vasodilation of arteries” (Table 3) or “cardiac dilation”, was observed in MWCNT exposed workers. In addition, the KEGG pathway enrichment analysis of down-regulated mRNAs, consistent with matched up-regulation of miRNAs, resulted in the enrichment of the “vascular smooth muscle- and cardiac muscle contraction”. This suggests that pulmonary exposure to MWCNTs might have the ability to decrease or inhibit muscle dilation in humans. The mRNA and miRNA expression changes upon exposure to MWCNTs in humans are well correlated with their ability to intensify or worsen pre-existing cardiovascular related diseases. A recent study by Erdely and Coworkers also demonstrated a close cross-talk between the pulmonary and systemic circulation, suggesting that a systemic response if chronic and persistent can initiate or exacerbate cardiovascular disease and dysfunction [64]. Most importantly the decreased expression of SOD1 and increased VCAM1 and IL6 levels were also reported by several epidemiological studies as potential biomarkers for medical surveillance of workers handling engineered nanomaterials [89, 90].

Identification and development of nanomaterial exposure-related biomarkers in the blood would be quite useful for monitoring as well as early diagnosis of various health effects in humans. Recently, miRNAs have attracted considerable attention as non-invasive biomarkers of a variety of human diseases (e.g., cancers), due to their remarkable stability and tolerance to RNase activity as well as extreme physiological conditions [91–94]. miRNAs are a family of small (~ 19–24 nucleotides long) non-coding functional RNAs that modulate gene expression of target mRNAs by binding to the 3′UTRs. Several studies also point towards their key role in regulating cellular processes and pathways that are critical for neoplastic transformation and tumor progression [93, 95–97]. The dysregulation of some miRNAs, referred to as “oncomiRs”, is associated with carcinogenesis and in promoting certain oncogenic events of cancer [98, 99]. A significant up-regulation of oncomiR mir-21-5p and miR-20a-5p (belonging to oncogenic miR-17-92 family or cluster) was found in the blood samples of MWCNT exposed workers (S1 File). The elevated expression of these miRNAs were reported in a variety of cancers including NSCLC and lung cancer [100] and are known to play roles in cell cycle regulation, apoptosis, cancer invasion and metastasis. Importantly, the different members of miR-17-92 cluster including miR-20a-5p –located on Chr13q31 –and mostly target genes involved in regulation and execution of G1/S transition, such as CCND1, E2Fs. The expression of both CCND1 and E2F2 was in fact found to be significantly down-regulated upon exposure to MWCNTs in this study (Fig 5A), further validating the increased levels of miR-20a-5p in our study and its regulatory role in targeting these genes (Fig 5A). The formation of CCND1-CDK4 complex is critical for regulating the cell-cycle during G(1)/S transition. Interestingly, the expression levels of mRNA coding for CDK4, in addition to CCND1, was also significantly decreased in MWCNT exposed workers. This suggests a cell cycle arrest at G1 phase, as elevated levels of CCND1–CDK4 complex promotes the transit from G0 to G1 during cell cycle, shortening the G1 cell cycle phase. This notion is further supported by the functional analysis studies of dysregulated mRNAs/miRNAs-mRNA target pairs performed in this study. The predicted top-ranked GSNs indicated CCND1 as the common hub gene exerting various biological functions with related roles in arrest in G1 phase, cell cycle progression, proliferation, G1/S phase transition and size/morphology of cells. Consistent with this, exposure to MWCNT bucky paper was shown previously to reduce the expression of CCND1/CDK4 as well as cell proliferation by triggering cell cycle arrest in G0/G1 phase and increase apoptosis in human leukemia cell lines through modulation of AKT and MAPK signaling pathways[59]. Similar results were also obtained on human normal peripheral blood lymphocytes where MWCNT bucky paper treatment blocked cell cycle progression by arresting cells at G0/G1 phase[101]. Especially, the enrichment of KEGG pathways including apoptosis, cell cycle regulation, PI3-Akt and MAPK by both up-/down-regulated mRNAs in the MWCNT exposed group further correlates MWCNT induced survival and apoptosis responses described previously (S3 & S4 Files). These findings suggest that MWCNTs are capable of exerting both cytostatic and cytotoxic effects in the whole blood of humans exposed to MWCNTs. In contrast to CCND1 & CDK4, a significant upregulation of another cyclin D member, CCND3 was observed upon MWCNT exposure. It has to be noted that the overexpression of CCND3 and down-regulation of CCND1 is further supported by a complex regulatory network involving noncoding miRNAs and lncRNAs (Fig 5). The up-regulation of CCND3 is further corroborated by the decreased levels of hsa-miR-16-5p in MWCNT-exposed workers (albeit with a p-value > 0.05). In addition to targeting CCND3, hsa-miR-16-5p is also known to regulate the expression of various angiogenic (e.g., PTGS2, VEGF, FGFR) and/or carcinogenic factors (e.g. EGFR, BMP7, MAPKs) with key roles in cancer/disease signaling pathways (Fig 5B). Most importantly, the overexpression of CCND3, probably to substitute for significantly decreased expression of CCND1 & CDK4, can result in dysregulation of the cell cycle and may further contribute to malignant phenotype of cells upon exposure to MWCNTs [102]. Some of these aberrantly expressed miRNAs are also located in chromosomal regions (e.g., chr13) frequently amplified in lung cancers [103]. Several *in vitro* studies also reported the potential of MWCNTs to induce inhibition of proliferation, cell cycle delay, apoptosis, malignant transformation, genotoxicity and abnormal chromosome number by interfering with mitosis (cell division) in various *in vitro* and *in vivo* studies [59, 101, 102, 104–111]. In fact, some of the miRNAs (e.g., ↑hsa-miR-20a-5p, ↑hsa-miR-21-5p, ↑hsa-miR-23a-5p, ↓hsa-miR-16-5p) and their mRNA targets aberrantly expressed in exposed workers are known to play critical roles in cell proliferation, apoptosis, tumor invasiveness and tumorigenesis as well as the pathogenesis of cancer including and NSCLC [103, 112–120]. Taken together, the results presented in this paper may suggest the onset of carcinogenic risk in humans potentially exposed to MWCNTs.

## Conclusions

In summary, we have performed a comparative analysis of ncRNA and mRNA expression profiles in non-exposed and workers exposed to MWCNT aerosols and provide important insights into the molecular details, pathways and regulatory networks of MWCNT-induced toxicity in humans. To the best of our knowledge this study is one of the first to investigate aberrant changes in mRNA and ncRNA expression profiles in the whole blood of humans exposed to MWCNTs. Identified changes in ncRNA and mRNAs, pathways and signaling networks revealed potential health risks and other outcomes associated with occupational exposures to MWCNTs. Although the aberrant changes in miRNAs, particularly in combination with their mRNA targets, have a promising potential as relevant blood markers for monitoring MWCNT exposure in humans, their potential prognostic value still needs further validation. It has to be noted that there are several limitations to this study including small sample size. A prospective large-scale study involving periodic collection of blood samples over longer latency periods and multiple CNT manufacturing locations along with detailed quantification of various cells in the blood and the role of confounding factors such as age, sex and smoking history, however, are required to further corroborate our findings. Such studies are underway and will support the potential applicability of the described blood markers to identify and monitor potential exposure and health risks associated with occupational exposure to MWCNTs.

## Supporting Information

S1 Fig Classification of CNT structures: matrix particles (A, B) and CNT cluster (C). (TIF)

S1 File Differentially regulated ncRNA and mRNA profiles in the high exposure group. The differentially up and down-regulated mRNA, lncRNA and miRNAs in the whole blood of workers from the high exposure group.
(XLSX)

S2 File Top ranked gene signaling networks based on mRNAs and miRNA-mRNA target pairs using IPA. The top 10 most significant GSNs generated based on mRNA and miRNA-mRNA target expression profiles in the blood of workers exposed to MWCNTs, along with significance scores, and their respective hub genes.
(XLSX)

S3 File KEGG pathway enrichment analysis of dysregulated mRNAs and miRNA-mRNA target pairs in the blood of the MWCNT high exposure group. The KEGG pathway enrichment analysis of up/downregulated mRNAs and mRNA targets of dysregulated miRNAs profiles were performed using DAVID[41] and DIANA-mirPath (ref), respectively. The prediction of mRNA target pairs corresponding to the up-regulated miRNAs was performed using both TargetScan and micro-TDS algorithms, provided by DIANA-miRPath. The KEGG pathways overrepresented by the mRNA target pairs of dysregulated miRNAs were cross compared to pathways enriched by downregulated mRNAs.
(XLSX)

S4 File The comprehensive list of all significantly dysregulated lncRNAs and their nearby coding genes (p-value<0.05, fold-change>±1.5) in the MWCNT high exposure group. The nearby coding genes identified based on their positional relationship to lncRNAs includes bidirectional, exon sense-overlapping, intergenic, intro sense-overlapping, intronic antisense and natural antisense relationships.
(XLSX)

S5 File The list of all aberrantly expressed miRNAs (fold-change>±1.5) and their corresponding interactions with dysregulated lncRNAs in the MWCNT high exposure group. The lncRNA–miRNA interaction dataset was downloaded with medium stringency from starBase v2.0 database [31, 32] in September, 2015. This dataset provides the most comprehensive list of experimentally confirmed and predicted lncRNA–miRNA interactions based on large scale CLIP-Seq data.
(XLSX)

S1 Table The physical and chemical properties of MWCNTs as reported by the manufacturing facilities investigated. (XLSX)

S2 Table Total CNT structure counts (CNT/mm2) for personal breathing zone samples, along with their maximum crosswise length distribution. Counts based on results (structures/grid openings) for three TEM grids.
(XLSX)

S3 Table Total and respirable elemental carbon (EC) concentrations of air samples collected in different technological and plant background areas of Nanotech center ltd. MWCNT facility (Tambov, Russia). (XLSX)

S4 Table Whole blood LncRNA expression profiles in humans exposed to MWCNT. Differentially expressed lncRNAs in the blood samples of workers non-exposed and potentially exposed to MWCNTs. (FC ≥ 1.5; *P*<0.05).
(XLSX)

S5 Table Whole blood mRNA expression profiles in humans exposed to MWCNT. Differentially expressed mRNAs in the blood samples of workers non-exposed and exposed to MWCNTs. (FC ≥ 1.5; *P*<0.05).
(XLSX)

S6 Table Disease association of dysregulated genes corresponding to mRNAs and miRNA-mRNA target pairs. The top five significantly (*P*< 0.05) up- and down- regulated genes corresponding to mRNAs and miRNA-mRNA target pairs of the overall and high exposure groups.
(XLSX)
