# Supplementary figures and images for: Integrated Analysis of Dysregulated ncRNA and mRNA Expression Profiles in Humans Exposed to Carbon Nanotubes
Source: PLoS One. 2016 Mar 1;11(3):e0150628. doi: 10.1371/journal.pone.0150628 (PMC4773015; doi:10.1371/journal.pone.0150628)

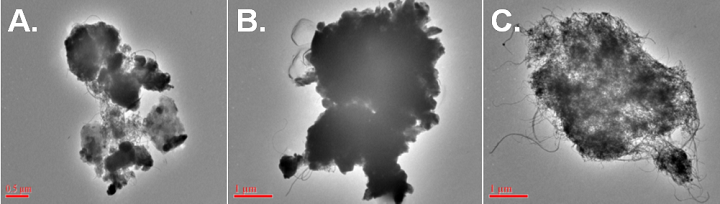

Supplement: S1 Fig — (TIF) [file pone.0150628.s001.tif]
